# Inhibition of ZEB1 leads to inversion of metastatic characteristics and restoration of paclitaxel sensitivity of chronic chemoresistant ovarian carcinoma cells

**DOI:** 10.18632/oncotarget.20107

**Published:** 2017-08-10

**Authors:** Jun Sakata, Fumi Utsumi, Shiro Suzuki, Kaoru Niimi, Eiko Yamamoto, Kiyosumi Shibata, Takeshi Senga, Fumitaka Kikkawa, Hiroaki Kajiyama

**Affiliations:** ^1^ Department of Obstetrics and Gynecology, Graduate School of Medicine, Nagoya University, Nagoya, Japan; ^2^ Department of Healthcare Administration, Graduate School of Medicine, Nagoya University, Nagoya, Japan; ^3^ Department of Obstetrics and Gynecology, Banbuntane Hotokukai, Fujita Health University, Fujita, Japan; ^4^ Division of Tumor Biology, Graduate School of Medicine, Nagoya University, Nagoya, Japan

**Keywords:** epithelial ovarian carcinoma, epithelial-mesenchymal transition, ZEB1, chemoresistance, metastasis

## Abstract

ZEB1, a member of the zinc-finger E-box binding homeobox family, is considered to play a crucial role in cancer progression and metastasis. In the current study, we investigated the role of ZEB1 in metastasis and chronic chemoresistance of epithelial ovarian carcinoma (EOC) cells. Using several EOC and acquired paclitaxel (PTX)-resistant EOC cell lines, we investigated whether silencing ZEB1 led to a reversal of the chemoresistance and metastatic potential *in vitro* and *in vivo*. Subsequently, the expression of ZEB1 in EOC tissues and its association with the oncologic outcome were investigated. According to the immunohistochemical staining of EOC tissues, as the positivity of ZEB1 expression was increased, the overall survival of EOC patients became poorer (*P* = 0.0022 for trend). Additionally, cell migration and invasion were significantly decreased by ZEB1 silencing in both PTX-sensitive and PTX- resistant cells. Although PTX-sensitivity was not changed by silencing ZEB1 in parental EOC cells, the depletion of ZEB1 made the PTX-resistant EOC cells more sensitive to PTX treatment. In an animal model, mice injected with ZEB1-silencing PTX-resistant cells survived for longer than the control cell-injected mice. Although the intravenous injection of PTX did not affect the tumor weight of shCtrl cells, the tumor weight of shZEB1 cells was significantly reduced by PTX treatment. The current data indicate the possible involvement of ZEB1 in the metastasis and paclitaxel resistance of EOC, and suggest that targeting this molecule may reverse the malignant potential and improve the oncologic outcome for EOC patients.

## INTRODUCTION

Epithelial ovarian carcinoma (EOC) is a major cause of mortality among all gynecological malignancies [1]. The recent Cancer Statistics estimated that 238,700 women were newly diagnosed with EOC, and 151,900 died of this tumor worldwide [2]. The prognosis of patients with EOC is most likely related to the degree of peritoneal dissemination [3–5]. Since EOC often remains silent in clinical situations, the majority of patients show aggressive peritoneal dissemination at the time of diagnosis [6]. Reflecting the cell biology of EOC, despite the fact that complete clinical remission can be achieved in approximately 80% of these patients owing to cytoreductive surgery, followed by systematic front-line chemotherapy, the majority of those clinical complete responders develop recurrent disease [7]. If all EOC tumor cells have sufficient sensitivity to conventional chemotherapy, they will disappear in due course. Nevertheless, since the sensitivity of tumors is actually heterogeneous, recurrence will eventually develop due to the remaining resistant tumor cells. EOC cells that have acquired chemoresistance may have hallmarks that favor easily spreading into the peritoneal cavity, resulting in an increased chance of adhering to the mesothelium and the enhanced formation of microscopic or macroscopic peritoneal metastasis *in vivo* [8]. Therefore, the clinical outcome of relapsed patients remains poor.

ZEB1, a member of the zinc-finger E-box binding homeobox (ZFH) family, is considered to play a crucial role in cancer progression and metastasis, it shows high-level expression in epithelial cancers, including prostate, hepatocellular carcinoma, lung, and pancreatic cancers, and its expression is correlated with a poor prognosis [9–11]. Through driving epithelial-mesenchymal transition (EMT), ZEB1 contributes to the metastasis of carcinoma cells, and prior studies demonstrated that ZEB1 conferred stemness and resistance [12]. Inhibition of ZEB1 reversed EMT and chemoresistance in chemoresistant human lung cancer cells [13]. In addition, interference with the ZEB1 function by the class I HDAC inhibitor mocetinostat led to the restoration of miR-203 expression, repressing stemness properties, and inducing sensitivity to chemotherapy [14]. Kikuchi et al demonstrated that Phenylbutyrate, a histone deacetylase antagonist that also exhibits antitumor activity sensitivity, was reported to be influenced by epigenetic expression alteration of ZEB1 in breast cancer cells [15]. This study proved that epigenetic regulation of ZEB1 may be a key biomarker for predicting resistance to breast cancer therapies. Furthermore, downregulation of ZEB1 by salinomycin increased the sensitivity of Mantle cell lymphoma cells to the cytotoxic effects of doxorubicin, cytarabine, and gemcitabine [16].

We previously demonstrated that chronic chemoresistance to paclitaxel (PTX) induced EMT and enhanced the peritoneal metastatic potential of EOC cells using a murine model [8]. Here, we aimed to clarify the role of ZEB1 in chemoresistance / metastasis, and clinical impact of ZEB1 expression in EOC by exploring: (i) ZEB1 expressions in various EOC cells and functions, including cell migration, invasion, and attachment to mesothelial cells, ii) ZEB1 expressions in two independent chronic PTX-resistant human EOC cell lines, which displayed a typical EMT phenotype, (iii) whether interfering ZEB1 expression restored sensitivity to PTX and exerted an anti-metastatic / chemoresistant potential, (iv) significance of ZEB1 expression in the peritoneal microenviroment displaying cell-to-cell communication between mesothelial and EOC cells, and (v) survival impact of ZEB1 expression in actual clinical samples. The possible function of the transcriptional factor as a facilitator of EOC metastasis is reported.

## RESULTS

### Expression of ZEB1 correlated with unfavorable outcome of patients with EOC

**Figure 1:**
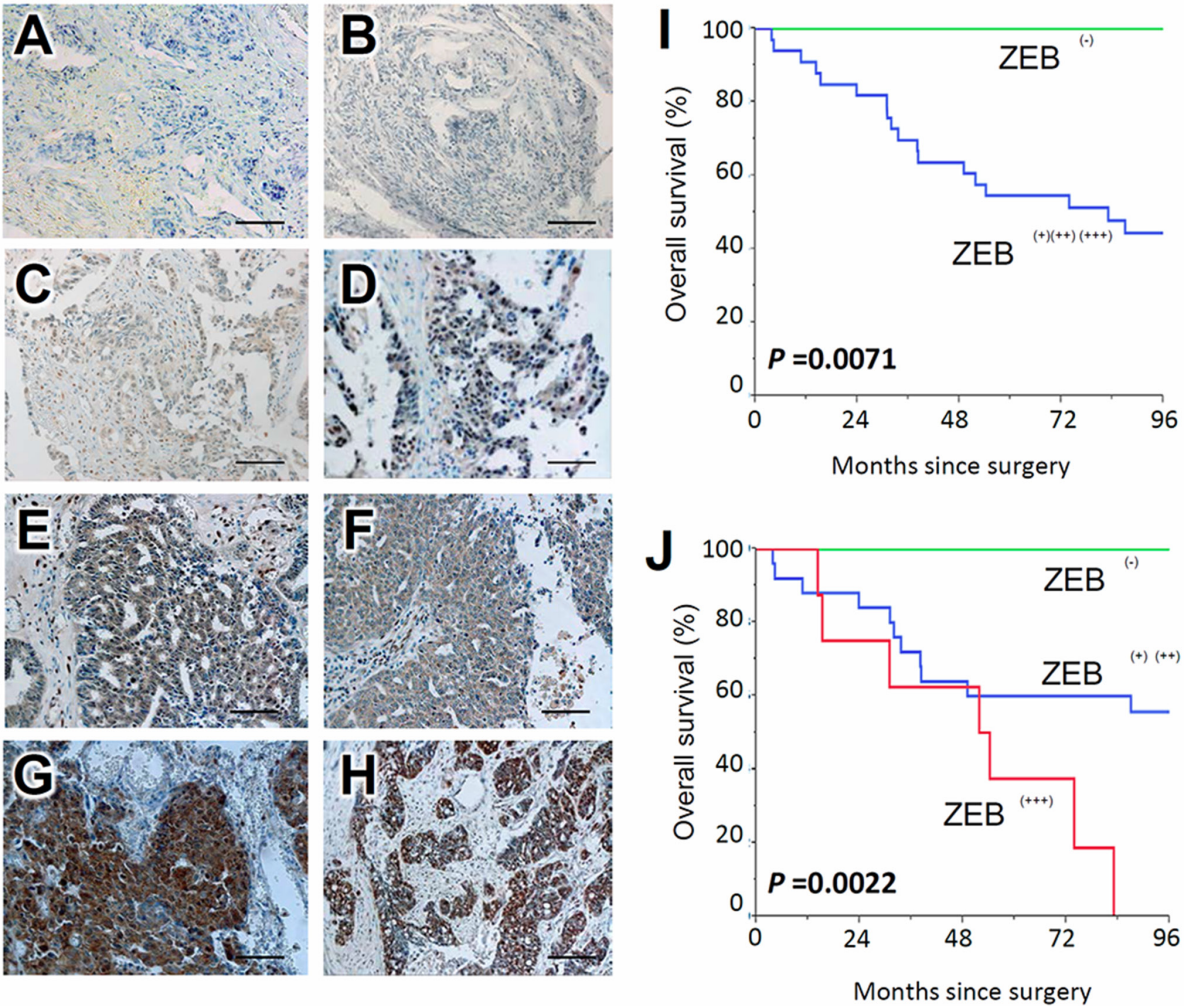
Survival impact of ZEB1 expression in EOC tissues Immunoreactivity of ZEB1 observed in surgical EOC samples (paraffin sections), positive or negative expression of ZEB1 in EOCs. (**A**, **B**) negative, (**C**, **D**) weakly positive, (**E**, **F**) moderately positive, (**G**, **H**) strongly positive; magnification × 100. (**I**, **J**) Kaplan-Meier overall survival curves for primary EOCs according to immunoexpression of ZEB1. Two-group comparison (I): Green line represents negative ZEB1 expression (negative: *N* = 7). Blue line represents positive ZEB1 immunoexpression (weekly-strongly positive: *N* = 33) (*P* = 0.0071). Three-group comparison (J): Green line represents negative ZEB1 expression (negative: *N* = 7). Blue line represents positive ZEB1 immunoexpression (weekly-moderately positive: *N* = 28). Red line represents positive ZEB1 immunoexpression (strongly positive: *N* = 8) (*P* = 0.0022).

The ZEB1 immunoreactivity was classified into the four scoring types as described in ‘‘Materials and Methods’’ (Negative, weakly, moderately, and strongly positive expressions). Representative images of each histological feature are shown in Figure 1A–1H.

In several cases, the immunoexpressions of ZEB1 were identified in the stroma as well as carcinoma tissues. Of the 40 carcinomas, negative, weakly, moderately, and strongly positive ZEB1 immunoexpressions were observed in 7 (17.5%), 14 (35.0%), 11 (27.5%), and 8 (20.0 %) patients, respectively. Compared with negative expression, positive ZEB1 expression predicted a significantly poorer overall survival {Negative vs. weak, moderate, and strong (*P* = 0.0071): Figure 1I}. Furthermore, as the positivity of ZEB1 expression was increased, the overall survival of EOC patients became poorer (*P* = 0.0022 for trend: Figure 1J).

### ZEB1 involved in migration, invasion, and adhesion to mesothelial cells, and PTX sensitivity of EOC cells

**Figure 2:**
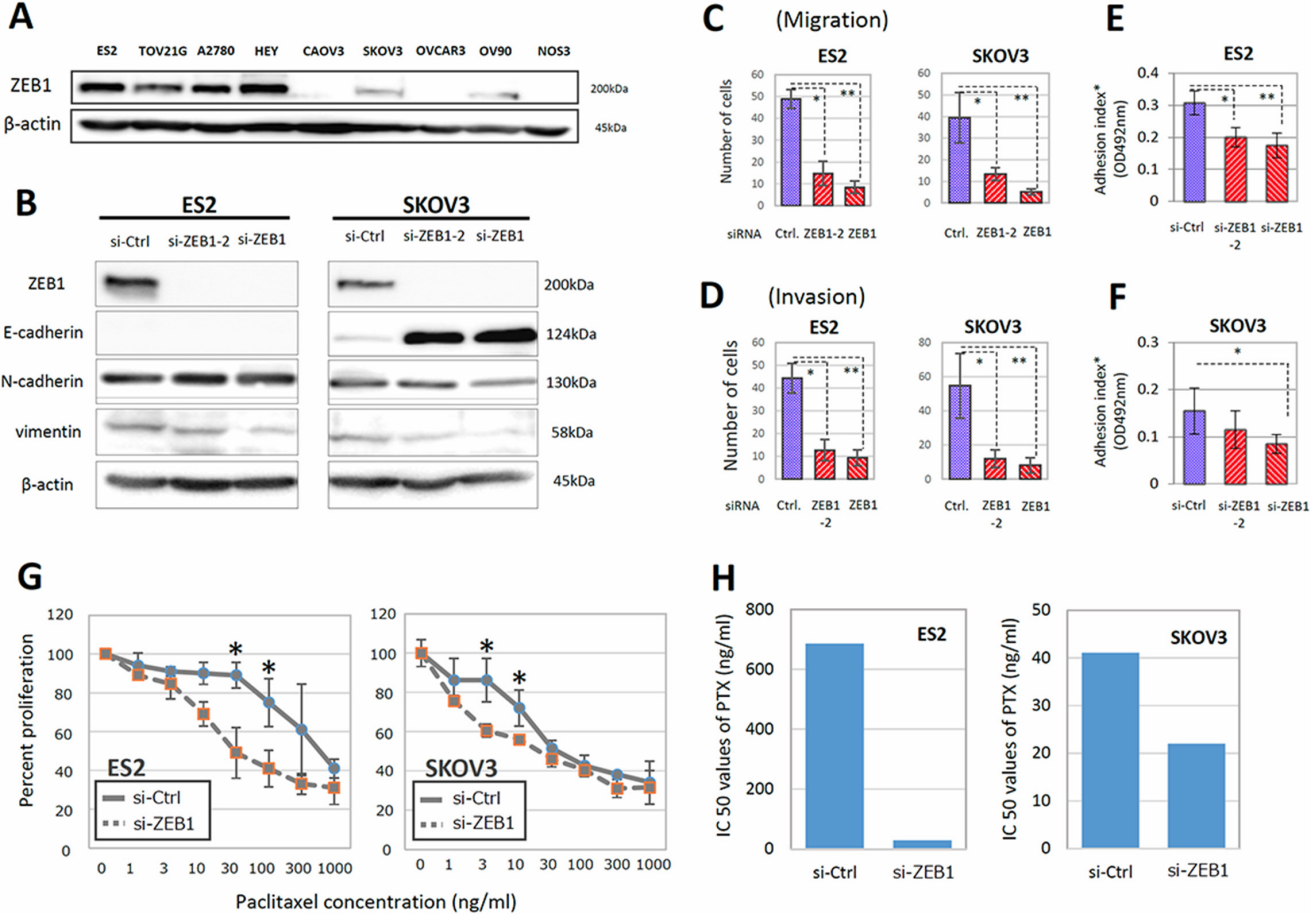
ZEB1 was involved in the migration, invasion, adhesion to mesothelial cells, and PTX sensitivity of EOC cells (**A**) The expression of ZEB1 in various EOC cells. ZEB1 was highly expressed in ES-2, TOV21G, A2780, and HEY cells. (**B**) Depletion of ZEB1 in ES-2 and SKOV3 cells and influence on expression of E-cadherin as an epithelial marker, and vimentin and N-cadherin as mesenchymal markers. (C, D) Effect of ZEB1 silencing on the migratory ability (**C**) and invasive potential (**D**) of ES-2 and SKOV3 cells. Asterisks show significance (*P* < 0.05). (**E**, **F**) Effect of ZEB1 silencing on the attachment of EOC cells to the confluent monolayer of human peritoneal mesothelial cells (HPMCs) (E) ES2 cells, (F) SKOV3 cells). Asterisks show significance (*P* < 0.05). (**G**, **H**) Restoration of PTX-resistance in ES-2 and SKOV3 cells by ZEB1 silencing. The PTX-sensitivity assay using evaluated by the MTT assay (G). Asterisks show significance (*P* < 0.05). ZEB1 knockdown clearly increased the sensitivities to PTX. IC50 values of PTX were significantly reduced by ZEB1 depletion in ES-2 and SKOV3 cells (H).

To investigate the role of ZEB1 in the malignant characteristics of EOC, we examined the expressions of ZEB1 in various EOC cells. ZEB1 was highly expressed in ES-2, TOV21G, A2780, and HEY cells. Moderate expression of ZEB1 was observed in SKOV3 and OV90 cells. We used ES-2 and SKOV3 cells, which showed high and moderate expression of ZEB1, respectively (Figure 2A). For further analyses, since ZEB1 is one of the major transcription factors that promote EMT in various cancer cells, we depleted ZEB1 in ES-2 and SKOV3 cells and examined the expression of E-cadherin as an epithelial marker, and vimentin and N-cadherin as mesenchymal markers. ZEB1 knockdown did not affect the expression of marker proteins in ES-2 cells; however, in SKOV3 cells, an increase of E-cadherin and decrease of vimentin and N-cadherin were clearly observed on ZEB1 depletion (Figure 2B). Similar tendencies were observed in TOV-21G, OV90, and A2780 cells (Supplementary Figure 1). These results are consistent with the previous finding that ZEB1 is associated with EMT.

EMT is often associated with increases in the migratory ability and invasive potential of cancer cells. To examine the migration of EOC cells, we used transwell chambers whose lower surface of the filter was coated with fibronectin. As shown in Figure 2C and Supplementary Figure 2A, the migration of both ES-2 and SKOV3 cells was significantly reduced by ZEB1 knockdown. To determine cell invasion, we used Matrigel-coated transwell chambers. ZEB1 depletion also suppressed the invasive potential of both cell lines (Figure 2D and Supplementary Figure 2B).

The peritoneal mesothelium is a monolayer of epithelial cells covering the peritoneal cavity and forming serosal membranes. The attachment of EOC cells to mesothelial cells is a critical step for cancer dissemination over the mesothelium. We tested if ZEB1 was required for the promotion of EOC cell attachment to HPMCs. ES-2 or SKOV3 cells transfected with siRNAs were seeded onto confluent monolayers of HPMCs and the attached cells were evaluated. As shown in Figure 2E, 2F, the suppression of ZEB1 reduced the attachment of both EOC lines to HPMCs. These results show that ZEB1 is required for cell migration, invasion, and attachment to mesothelial cells.

We subsequently examined whether ZEB1 was associated with the acquisition of PTX-resistance *in vitro*. ES-2 and SKOV3 cells transfected with siRNAs were treated with different concentrations of PTX, and the surviving cells were evaluated by the MTT assay, as described in ‘‘Materials and Methods’’. In both lines, ZEB1 knockdown clearly increased the sensitivities to paclitaxel. The IC50 values of PTX were significantly reduced by ZEB1 depletion in both lines (Figure 2G, 2H).

### ZEB1 expression and its function in chronic PTX-resistant EOC cells

**Figure 3:**
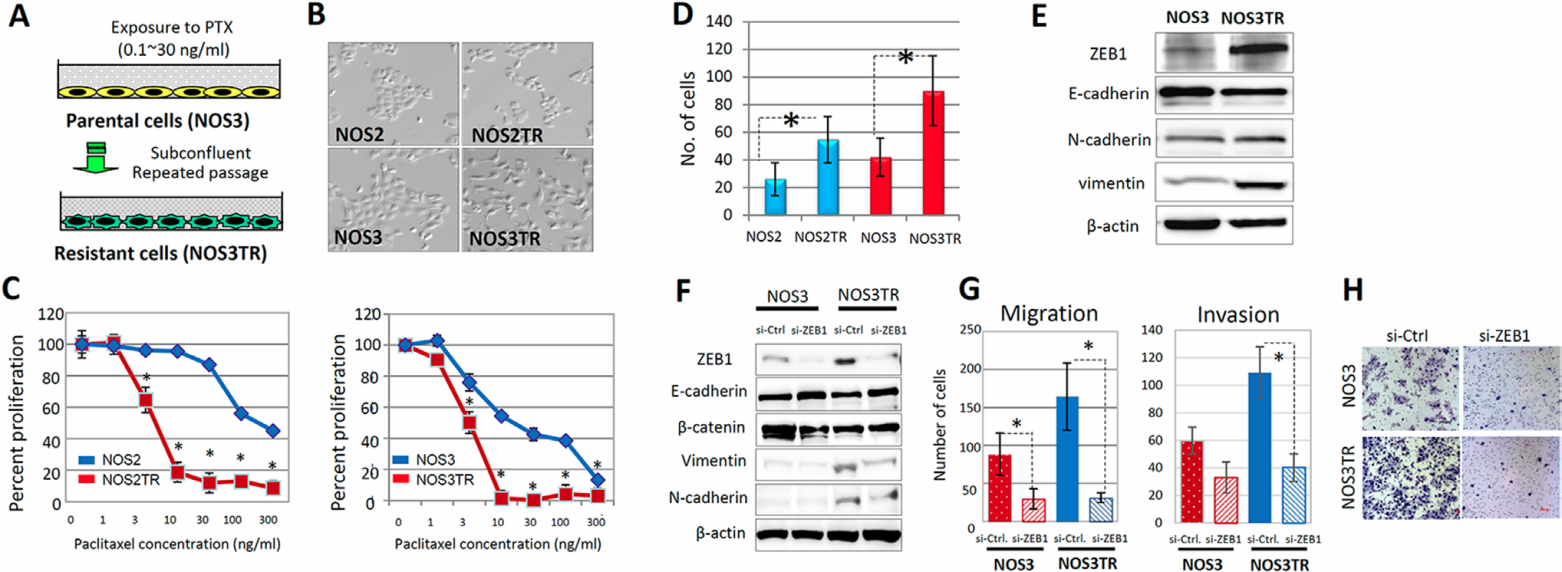
ZEB1 expression and function in chronic PTX-resistant EOC cells (**A**) Generation of two independent PTX-resistant cell lines using NOS2 and NOS3 cells by continuous exposure to stepwisely increasing concentrations of PTX. (**B**) The morphology of two independent PTX-resistant cell lines (NOS2TR and NOS3TR cells: spindle-shaped). (**C**) PTX-sensitivity assay of the chronic PTX-resistant cells using evaluated by the MTT assay. ZEB1 knockdown clearly increased sensitivities to PTX. Asterisks show significance (*P* < 0.05). (**D**) The migratory potentials of NOS2TR and NOS3TR cells. Asterisks show significance (*P* < 0.05). (**E**) Western blot analysis showing the increased expression of ZEB1 and induced mesenchymal characteristics in acquired PTX-resistant cells (NOS3TR). (**F**) Western blot analysis: Induction of enhanced expression of E-cadherin and downregulation of vimentin by ZEB1 knockdown. (**G**) The effect of ZEB1 silencing on cell migration and invasion of NOS3 and NOS3TR cells. Asterisks show significance (*P* < 0.05). (**H**) Representative images of the decreased cell migration observed between NOS3 and NOS3TR cells.

To further confirm that ZEB1 was required for chemoresistance, we generated two independent PTX-resistant cell lines using parental NOS2 and NOS3 cells, which showed low expression of ZEB1. We treated both cell lines for months with different concentrations of PTX and finally obtained highly PTX-resistant cells, NOS2TR and NOS3TR, respectively (Figure 3A). The morphology of NOS2TR and NOS3TR cells became spindle-shaped with disrupted cell-cell adhesion (Figure 3B). Nearly 80–100% of NOS2 and NOS3 cells died in the presence of 10 ng/mL of PTX, whereas approximately 40% of both lines were alive with 100 ng/mL of PTX (Figure 3C). NOS2TR and NOS3TR showed increased cell migration and invasion, compared with each parental cells line (Figure 3D). In addition, we examined whether chronic PTX-resistant cells showed the mesenchymal characteristics by immunoblot. As shown in Figure 3E, the expression of E-cadherin was decreased and a clear increase of vimentin was observed. Consistent with the enhanced migratory potential, ZEB1 expression was higher in NOS3TR cells than parental NOS3 cells. Furthermore, we examined the effect of silencing ZEB1 on both migration and invasion of chronic PTX-resistant EOC cells. As shown in Figure 3F, ZEB1 knockdown induced the enhanced expression of E-cadherin and downregulation of vimentin. Accordingly, migration and invasion were significantly decreased by ZEB1 silencing in NOS3TR cells (Figure 3G). A representative image of decreased cell migration is presented in Figure 3H.

We also examined whether ZEB1 was required for the promotion of chronic PTX-resistant cell attachment to HPMCs. NOS3 and NOS3TR cells transfected with siRNAs were seeded onto the confluent monolayer of HPMCs and attached cells were evaluated. As shown in Supplementary Figure 3, suppression of ZEB1 significantly reduced the attachment of NOS3TR cells to HPMCs, although it did not affect NOS3 cells.

### Restoration of PTX-sensitivity by silencing ZEB1 expression in chronic PTX-resistant cells

**Figure 4:**
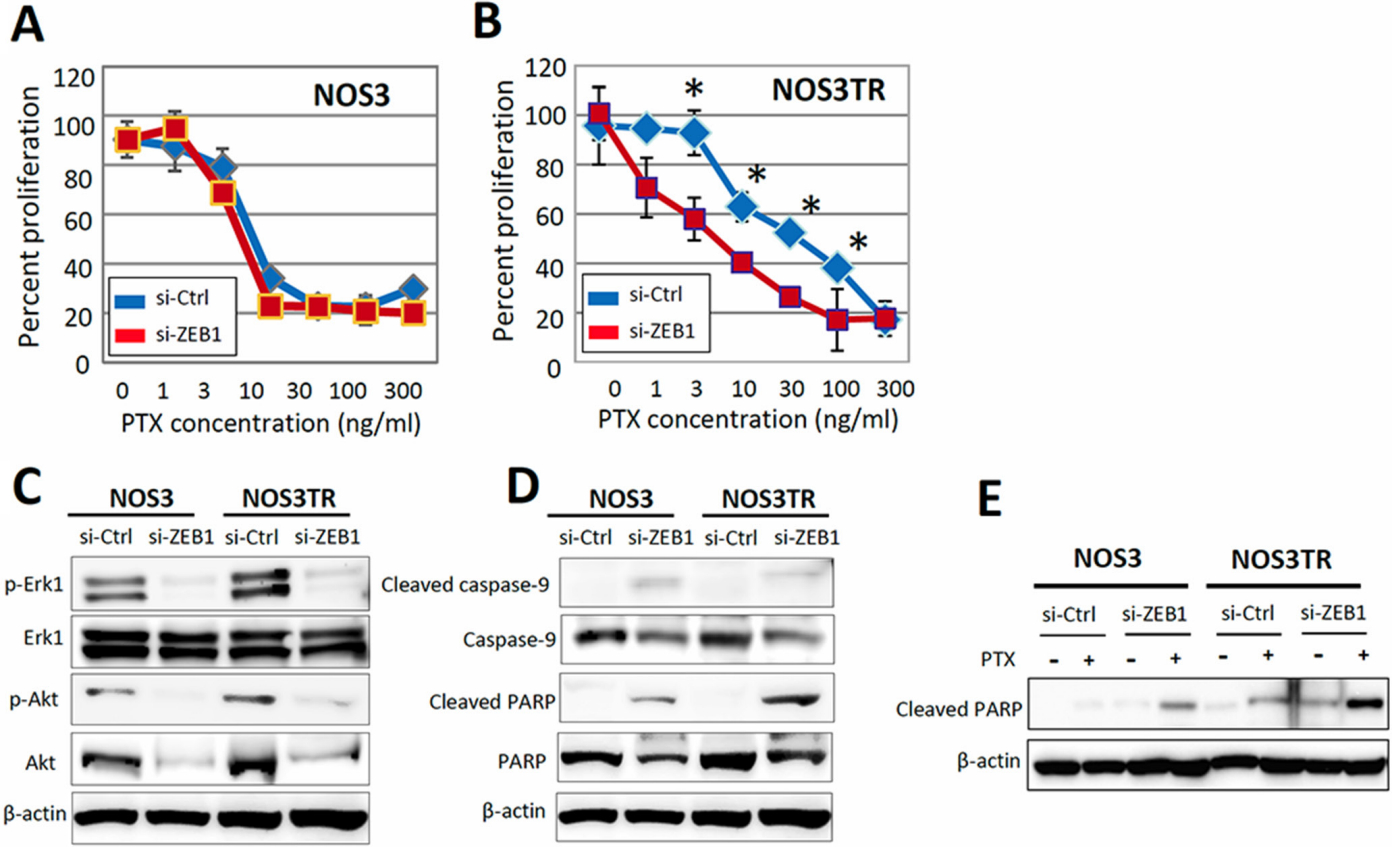
Restoration of PTX-sensitivity by silencing ZEB1 expression in chronic PTX-resistant EOC cells (**A**) The PTX-sensitivity curves with or without silencing of ZEB1 in parental NOS3 cells. Asterisks show significance (*P* < 0.05). (**B**) The PTX-sensitivity curves with or without silencing of ZEB1 in parental NOS3TR cells. Depletion of ZEB1 made NOS3TR cells more sensitive to PTX treatment. Asterisks show significance (*P* < 0.05). (**C**) The expression of phosphorylation of ERK1 and Akt. The phosphorylation of ERK1 and Akt was significantly decreased in ZEB1-depleted NOS3TR cells compared with that of control siRNA-transfected NOS3TR cells. (**D**) The greater induction of cleaved PARP expression in ZEB1-depleted NOS3TR cells compared with that of NOS3 cells. (**E**) Significantly higher expression of cleaved PARP in ZEB1-depleted NOS3TR cells in the presence of PTX, compared with that of control siRNA-transfected NOS3TR cells.

Next, we investigated whether the acquired PTX-resistance of NOS3TR was dependent on the increased expression of ZEB1. In parental NOS3 cells, the PTX-sensitivity was not changed by silencing of ZEB1 (Figure 4A). In contrast, the depletion of ZEB1 made NOS3TR cells more sensitive to PTX treatment (Figure 4B). The IC50 value of control siRNA-transfected NOS3TR cells was 41.8 ng/mL, whereas that of ZEB1-depleted NOS3TR was 6.2 ng/mL. To further clarify the mechanism of PTX-sensitivity by ZEB1 knockdown, we examined the phosphorylation of ERK1 and Akt. As shown in Figure 4C, the phosphorylation of ERK1 and Akt was significantly decreased in ZEB1-depleted NOS3TR cells compared with that of control siRNA-transfected NOS3TR cells. However, this reduction was also observed in parental NOS3 cells. In addition, we examined the expression of cleaved PARP, which is an indicator of the induction of apoptosis. As shown in Figure 4D, the induction of cleaved PARP was more markedly observed in ZEB1-depleted NOS3TR cells compared with NOS3 cells. Furthermore, in the presence of PTX, the expression of cleaved PARP was significantly higher in ZEB1-depleted NOS3TR cells compared with that of control siRNA-transfected NOS3TR cells (Figure 4E).

### Enhancement of ZEB1 expression by the microenvironmental production of TGF-β

**Figure 5:**
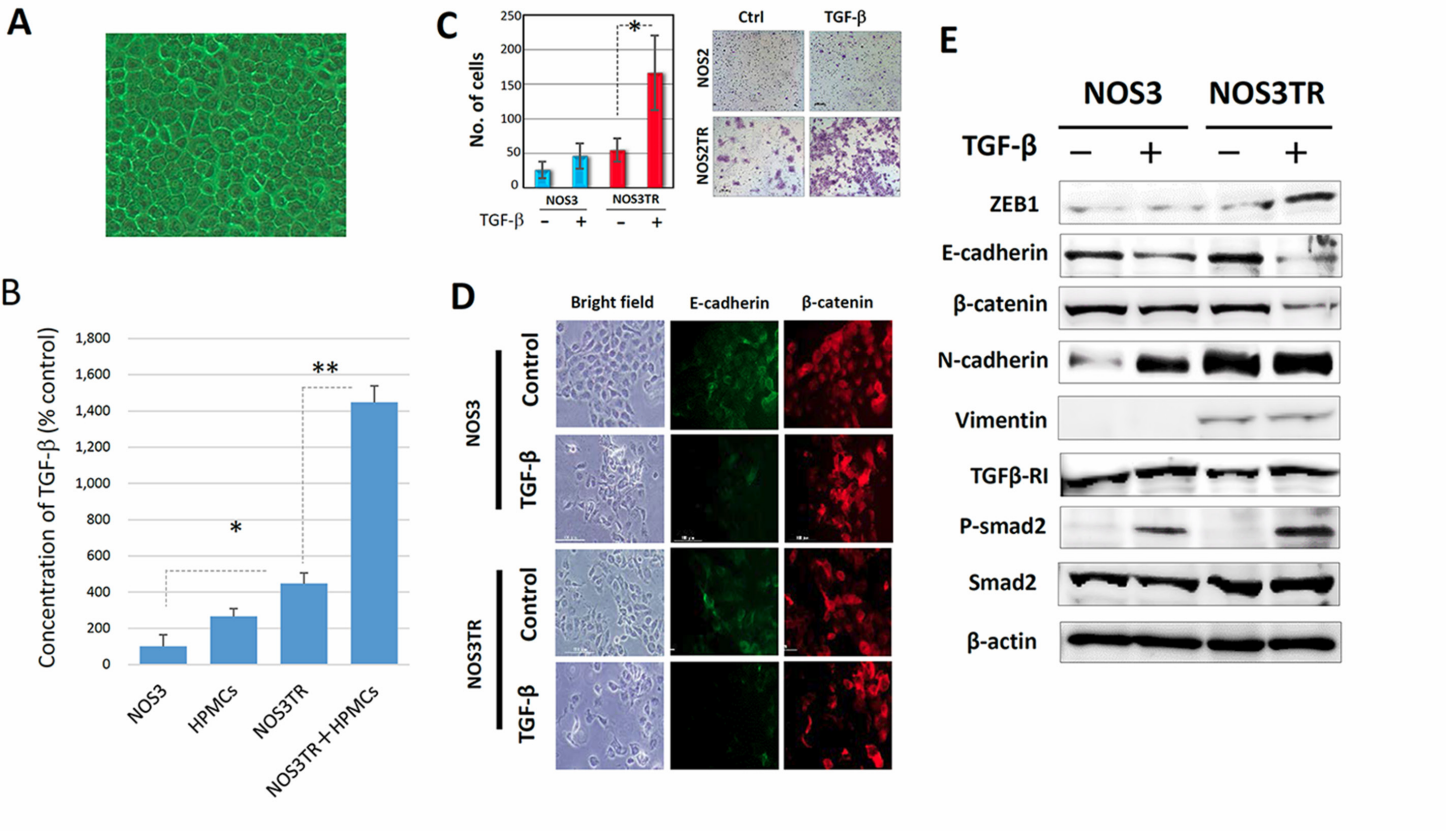
Enhanced ZEB1 expression by microenvironmental production of TGF-β (**A**) The appearance of the cultured human peritoneal mesothelial cells (HPMCs). HPMCs typically showed an epithelial morphology with a cobblestone appearance. (**B**) TGF-β concentrations in conditioned media of HPMCs, NOS3, NOS3TR, and HPMCs plus NOS3TR cells (co-culture). TGF-β production in NOS3TR cells was significantly higher than in parental NOS3 cells. Asterisks show significance (*P* < 0.05). (**C**) The effect of TGF-β on migratory potential of parental or PTX-resistant cells. The addition of TGF-β to NOS3TR cells led to a significantly enhanced migratory potential compared with parental NOS3 cells (Right panel). Asterisks show significance (*P* < 0.05). Left panel: representative images. (**D**) The immunofluorescence expressions of E-cadherin and β-catenin in NOS3 or NOS3TR cells with or without TGF-β. The presence of TGF-β caused a more mesenchymal cell shape with the loss of cell polarity and decreased E-cadherin expression, particularly in NOS3TR cells. (**E**) Western blot analysis of the EMT, or TGF-β signal-related proteins in NOS3 and NOS3TR cells with or without TGF-β. The addition of TGF-β resulted in increased ZEB1 expression with the enhanced expression of phosphorylated Smad 2, particularly in NOS3TR cells.

As previously mentioned, HPMCs serve as a protective barrier, being the front-line cellular barrier against disseminated EOC cells [17]. HPMCs typically shows an epithelial morphology with a cobblestone appearance (Figure 5A). TGF-β has been identified as a representative inducer of EMT. We initially examined the TGF-β concentration in conditioned media of HPMCs, NOS3, NOS3TR, and HPMCs plus NOS3TR cells (co-culture condition). As shown in Figure 5B, TGF-β production in NOS3TR cells was significantly higher than in parental NOS3 cells. However, it was synergistically higher under the co-culture condition with HPMCs. We subsequently tested the effect of TGF-β in EMT-related characteristics in PTX-resistant NOS3TR cells. As a result, the addition of TGF-β to NOS3TR cells led to a significantly enhanced migratory potential compared with parental NOS3 cells (Figure 5C). In addition, the presence of TGF-β led to a more mesenchymal cell shape with the loss of cell polarity and decreased E-cadherin expressions, particularly in NOS3TR cells (Figure 5D). Furthermore, we subsequently examined whether the EMT, or TGF-β signal-related proteins were altered in NOS3 and NOS3TR cells with or without TGF-β. The addition of TGF-β resulted in increased ZEB1 expression with the enhanced expression of phosphorylated Smad2, particularly in NOS3TR cells (Figure 5E). Those results suggest that TGF-β produced in the microenvironmental cell-to-cell communication contributes to the upregulation of ZEB1, thought to play a role in chronic PTX-resistance/peritoneal metastasis.

### ZEB1 required for chemo-resistance to PTX *in vivo*

**Figure 6:**
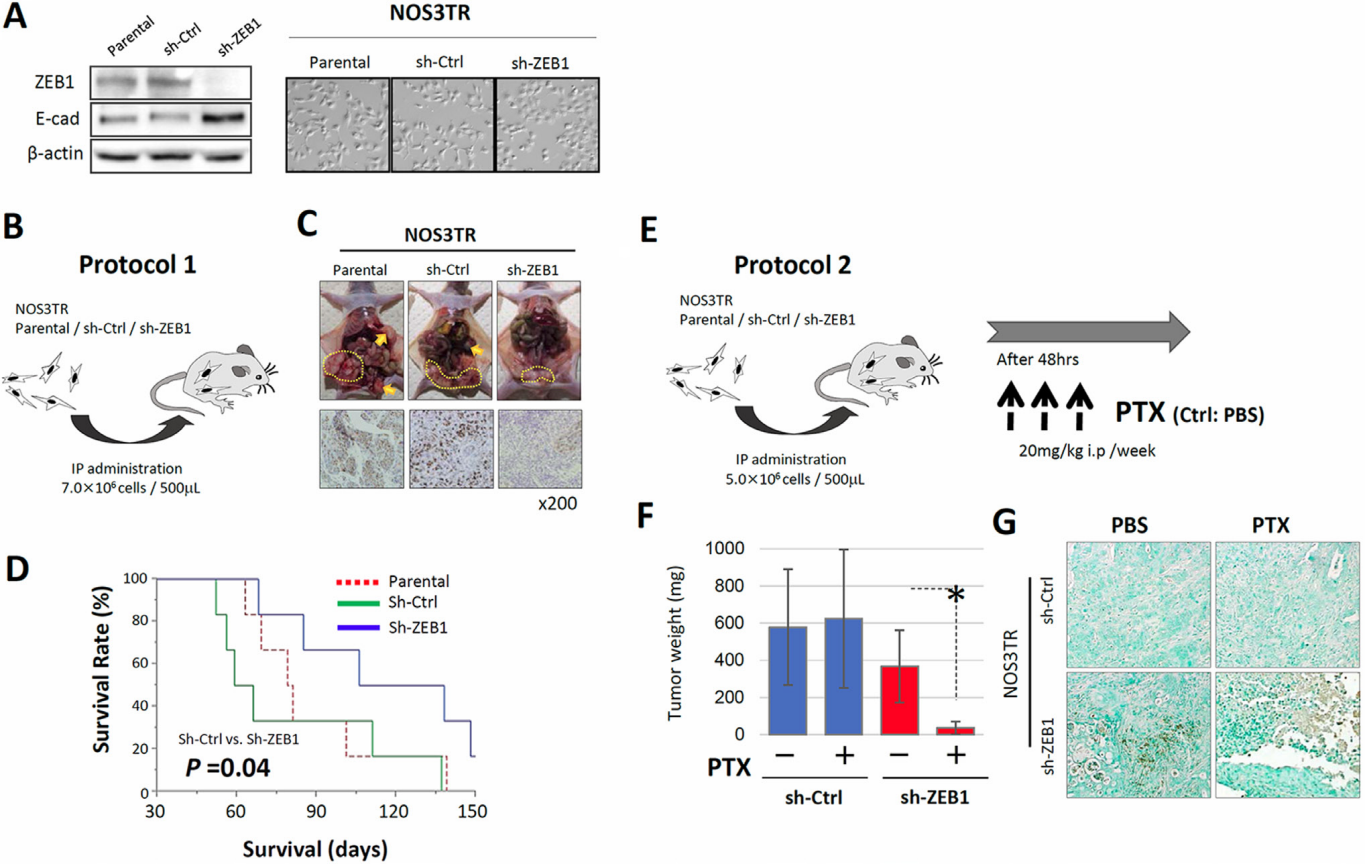
ZEB1 is required for chemo-resistance to PTX *in vivo* (**A**) Generation of NOS3TR cells that constitutively expressed control shRNA (shCtrl) or ZEB1 shRNA (shZEB1) by retrovirus infection (immunoblot). The morphology of shCtrl and shZEB1 cells. shZEB1 showed an epithelial cell-like morphology with clear cell-cell adhesion. (**B**) The injection of either shCtrl or shZEB1 cells into the murine peritoneum (IP administration: 7.0 × 10^6^ cells / 500 μL). (**C**) Decreased intraperitoneal dissemination in the shZEB1 cell-inoculated mice, compared with the control mice (upper panels). Suppressed expressions of ZEB1 observed in the peritoneal metastatic lesions in the shZEB1 cell-injected mice (lower panels). (**D**) Survival curves of the parental, shCtrl or shZEB1 cell- inoculated mice. The mice injected with shZEB1 cells survived for longer than the shCtrl-injected mice (*P* = 0.04). (**E**) The *in vivo* PTX-sensitivity model. Mice were intraperitoneally injected with either shCtrl or shZEB1 cells, and were treated with or without 20 mg/kg of PTX once a week for three weeks. Mice were sacrificed 4 weeks after tumor cell injection and the total volume of disseminated tumors of each mouse was measured. (**F**) Total weight of intraperitoneal disseminated tumors in shCtrl and shZEB1 cell-inoculated mice in the presence or absence of PTX treatment. Asterisks show significance (*P* < 0.05). (**G**) TUNEL assay of tumor tissues from PTX-treated mice. A number of shZEB1 cells were apoptotic, whereas only a small fraction of shCtrl cells were positive for apoptosis.

To further extend our analysis, we performed *in vivo* analyses using nude mice. We generated NOS3TR cells that constitutively expressed control shRNA (shCtrl) or ZEB1 shRNA (shZEB1) by retrovirus infection. Clear reduction of ZEB1 expression in shZEB1 cells was observed by immunoblot. Consistent with the reduction of ZEB1 expression, shZEB1 showed an epithelial cell-like morphology with tight cell-cell adhesion (Figure 6A). After either shCtrl or shZEB1 cells had been injected into the murine peritoneum, the dissemination of cancer cells on the mesothelium was examined (Figure 6B). As shown in Figure 6C, the proliferation of shZEB1 cells in peritoneum was suppressed compared with shCtrl cells. We confirmed that the immunoexpression of ZEB1 was lower in shZEB1-cells-formed tumors than those of shCtrl cells. In addition, metastasis to other organs, such as the pancreas, was significantly suppressed by ZEB1 knockdown (Table 1). In accordance with the reduced proliferation and metastasis, mice injected with shZEB1 cells survived for longer than shCtrl-injected mice (Figure 6D).

**Table 1:** The frequency of metastasis in each organ in the absence or presence of the PTX treatment

Metastasis	NOS3TR sh-Ctrl			NOS3TR sh-ZEB1		
	PBS	PTX	(Total)	PBS	PTX	(Total)
**Diaphragm**	3/4^*^	2/4	(5/8)	0/4^*^	0/4	(0/8)
**Liver**	3/4	2/4	(5/8)	2/4	1/4	(3/8)
**Pancreas**	3/4^**^	4/4^***^	(7/8)	0/4^**^	0/4^***^	(0/8)
**Spleen**	0/4	1/4	(1/8)	0/4	0/4	(0/8)

We finally investigated whether shZEB1 cells were more sensitive to PTX *in vivo*. Mice were intraperitoneally injected with either shCtrl or shZEB1 cells and were non-treated or treated with 20 mg/kg of PTX once a week for three weeks (Figure 6E). Mice were sacrificed 4 weeks after tumor cell injection and the total weight of disseminated tumors of each mouse was measured. Although the intravenous injection of PTX did not affect the tumor weight of shCtrl cells, tumor weight of shZEB1 cells was significantly reduced by PTX treatment (Figure 6F). The Tunnel assay of tumor tissues from PTX-treated mice showed that a number of shZEB1 cells were apoptotic, whereas only a small fraction of shCtrl cells were positive for apoptosis (Figure 6G). Table 1 shows the frequency of metastasis in each organ in the absence or presence of PTX treatment. Regardless of PTX treatment, recurrences in the diaphragm and pancreas were more frequently observed in the shZEB1-inoculated group than the Ctrl-inoculated group. These results indicate the pivotal role of ZEB1 in the acquisition of resistance to PTX *in vivo*.

## DISCUSSION

Peritoneal metastasis is the most frequent clinical presentation of EOC, composed of multiple steps: release from the original ovarian neoplasm, attachment to the mesothelium, and subsequent migration/invasion into the subperitoneal tissue. Although most EOC patients with peritoneal dissemination are asymptomatic until the late stage, tumor dissemination throughout the peritoneal cavity gradually spreads underneath the surface. On the other hand, as well as peritoneal metastasis, intrinsic or acquired chemoresistance remains a major therapeutic hurdle to improve the prognosis of patients with EOC. PTX, which exerts its effect through the stabilization of microtubules, induction of cell cycle arrest in G2-M, and activation of proapoptotic signaling, is a first-line chemotherapeutic agent that is effective for the treatment of EOC [8, 18]. Although PTX as a front-line antineoplastic agent has contributed to improve the short-term survival, the 5-year survival rate is lower than 30% in advanced EOC patients [19]. In clinical practice we occasionally encounter sudden recurrences in the peritoneal cavity or lymph nodes during intermittent treatment with antineoplastic drugs, including PTX. The increasing metastatic potential and process of chemoresistance may have concurrently developed during the evolution of tumors toward increasingly malignant characteristics. Considering such molecular mechanisms, we previously revealed that chronic PTX-resistant EOC cells displayed more mesenchymal features, such as the loss of the epithelial phenotype, acquisition of fibroblast-like morphology, switch of EMT-related markers, and enhanced migratory potentials both *in vitro* and *in vivo* [8]. However, detailed molecular mechanisms underlying such chemoresistance and subsequent metastasis-promoting potential in EOC have yet to be clarified. In our current analysis, we focused on ZEB1, which is one of the major transcription factors that facilitate EMT and its expression was reported to be associated with a poor clinical outcome in patients with several solid malignancies [9–11].

We first demonstrated that various expression levels of ZEB1 were identified in 82.5% of the EOC tissue samples. Regarding the correlation between the ZEB1 expression and prognosis, positive ZEB1 expression was a significant predictor of poorer overall survival compared with negative expression. Furthermore, as the positivity of ZEB1 expression incresed, patients accordingly showed poorer overall survival. Earlier studies revealed that the positive expression of ZEB1 in endometrial cancers, colorectal carcinomas, and hepatocellular and prostate cancer was associated with aggressive disease, poor differentiation, the development of metastases, and a poor clinical prognosis [20–22]. Our current findings are consistent with such prior evidence. Therefore, we could obtain evidence that ZEB1 plays a central role in the progression of EOC. A possible explanation for this finding was that an unfavorable outcome in patients with ZEB1 may be due to the chemoresistance and increased metastatic ability of EOC.

We subsequently examined the molecular mechanism whereby ZEB1 exerted its tumor-promoting effect in EOC *in vitro*. Using various EOC cell lines, we found that ZEB1 was expressed in highly metastatic EOC cell lines, and the depletion of ZEB1 expression led to downregulation of the migration, invasion, adhesion to mesothelial cells, and PTX sensitivity of EOC cells. Therefore, the results showing that an increase of E-cadherin and decrease of vimentin and N-cadherin were clearly observed by ZEB1 depletion were consistent with the occurrence of mesenchymal-epithelial transition. As aforementioned, one of the major therapeutic barriers to improving such a poor clinical outcome is chemoresistance that is related to a multi-network of molecular mechanisms [23]. In the current examination, the knockdown of ZEB1 significantly restored PTX-sensitivity and reduced motility / invasiveness in chemoresistant cells, suggesting that ZEB1 contributes to the development / maintenance of both the PTX-resistance and metastatic potential of these cells. In other words, ZEB1 may also switch the EOC cell phenotype from non-metastatic to highly metastatic and from chemosensitive to chemoresistant. According to a previous report, ZEB1 was associated with cell sensitivity to conventional chemotherapy, including gemcitabine, 5-fluorouracil (5-FU), and cisplatin in cancer cells [24]. In addition, silencing ZEB1 expression could significantly restore the chemosensitivity of docetaxel-resistant human lung adenocarcinoma cells as well as inhibit their migratory ability through reversing the mesenchymal phenotype [13]. Our current findings are consistent with such earlier evidence. Accordingly, we could reveal that ZEB1 plays a pivotal role in the acquired chemoresistance of EOC cells.

The TGF-β signaling pathway is one of the pivotal regulators of the multistep tumor microenvironment related to EMT [25–27]. Similarly to other EMT-inducible markers, such as Snail, Twist, and Slug, ZEB1 was significantly enhanced by the stimulation of TGF-β [28]. The interaction between the peritoneal mesothelium and tumor cells plays a major role in ovarian cancer progression. Our data showed that TGF-β was produced by EOC cells and it was synergistically upregulated under co-culture conditions with human normal mesothelial cells. Actually, it is plausible that TGF-β, which is derived from tumor cells, is accumulated in the peritoneal cavity via malignant ascites, and influences mesothelial cells. In our earlier study, an increase of exogenous TGF-β induced the additional production of endogenous TGF-β from mesothelial cells, resulting in the synergistic accumulation of TGF-β [29]. Interestingly, in the current study, TGF-β production in the chronic PTX-resistant cells was significantly higher than in naive EOC cells. In addition, the TGF-β stimulation led to the synergistic increase of ZEB1 expression with the enhanced expression of phosphorylated Smad 2, particularly in the PTX-resistant cells. These results suggest that the microenvironmental cell-to-cell communication between EOC and the mesothelium contributes to the auto-stimulatory TGF-β accumulation and corresponding increase of ZEB1, resulting in the further acquisition of the metastatic potential and/or chronic PTX-resistance of EOC. However, our current data were too preliminary and hypothesis-generating, to understand the whole picture of TGF-β/ZEB1 axis in EOC behavior in the peritoneal cavity. We would like to clarify it through further examinations in a future study.

In summary, these findings suggest that ZEB1 functions as both a chemoresistance- and metastasis-promoting factor in EOC. The overexpression of ZEB1 was found in EOC tissues and chemoresistant EOC cell lines. This is the first report showing that ZEB1 knockdown can enhance the sensitivity of EOC cells to PTX as well as the metastatic potential related to EMT. Furthermore, our data suggest a possible association between the PTX-resistance and enhanced metastatic potential of EOC through the ZEB1-TGF-β axis. In our animal model, in accordance with the reduced proliferation and metastasis, mice injected with ZEB1 silencing PTX-resistant EOC cells survived for longer than the control cell-injected mice. Although the current findings must be confirmed by other future studies, ZEB1 may be a helpful biological marker and possible target for EOC therapy. Thus, ZEB1 should be explored as a candidate therapeutic target for modulating the paclitaxel sensitivity of EOC. However, one of the most important prognostic factors for EOC is thought to be the resistance to cisplatin. To our knowledge, the association of ZEB1 expression with the cisplatin-resistance has not been clarified in the literature. Unfortunately, we cannot examine this topic in the current study. We would like to elucidate it in the future. At least, the current findings indicate that the immunoreactive identification of ZEB1 expression might be a crucial predictor of patients who will show a poor oncologic outcome, and its identification may lead to the selection of better treatment strategies.

## MATERIALS AND METHODS

### Cell culture

The ES-2, TOV21G, A2780, HEY, SKOV3, OVCAR3, CAOV3, and OV90 cell lines were maintained in RPMI1640 medium with 10% FBS and penicillin/streptomycin. These cell lines were obtained from the American Type Culture Collection (Manassas, VA, USA) in 2012–2013. The NOS2 and NOS3 cells, derived from serous EOC, were established in our institute [30, 31]. These cell lines were maintained in RPMI-1640 (Sigma, St. Louis, MO, USA) supplemented with 10% fetal bovine serum (FBS) and penicillin-streptomycin at 37°C in a humidified atmosphere of 5% CO_2_. The NOS2TR and NOS3TR cells, established from parental NOS2 and NOS3 cells, respectively acquired chronic resistance to paclitaxel [32–34]. Human peritoneal mesothelial cells (HPMCs) were isolated from surgical specimens of the human omentum after obtaining consent from each patient and approval of the Ethics Committee of Nagoya University. The procedure for HPMC isolation was described in detail in our previous report [35]. HPMCs were cultured in medium composed of RPMI-1640 (Sigma, St. Louis, MO, USA) supplemented with 10% FBS and penicillin-streptomycin.

### Immunohistochemical staining

Tissue samples of EOC were obtained after receiving informed consent from patients who were surgically treated at Nagoya University Hospital. Formalin-fixed, paraffin-embedded tissue sections were cut at a thickness of 4 μm. For heat-induced epitope retrieval, deparaffinized sections in 0.01 M citrate buffer (Target Retrieval Solution, pH 6.1, Dako) were heated three times at 90°C for 5 min using a microwave oven. Immunohistochemical staining was performed using the avidin–biotin immunoperoxidase technique with the Histofine SAB-PO kit (Nichirei, Tokyo, Japan) according to the manufacturer’s protocol, and the detailed experimental procedure was described previously [36]. Sections were incubated at 4°C for 12 hrs with primary antibody (anti-rabbit- ZEB1 polyclonal, at a 1: 1,000 dilution, Cell Signaling, MA, USA). As a negative control, the primary antibody was replaced with normal rabbit IgG at an appropriate dilution. The intensity of immunostaining for ZEB1 was scored semiquantitatively on a four-tiered scale based on the percent positivity / area of stained cells as follows: For the evaluation of ZEB1 expression, the staining intensity was scored as 0 (negative), 1 (weak), 2 (medium), or 3 (strong). The extent of staining was scored as 0 (0 %), 1 (1 %–10 %), 2 (10 %–50 %), or 3 (51 % <) according to the percentage of the positive staining area relative to the total tumor area. The sum of the intensity and extent scores was used as the final staining score (0–6) for ZEB1. Tumors with a final staining score of 0, 1–2, 3–4, and 5–6 were considered to have negative, weakly, moderately, and strongly positive expression, respectively.

### Inhibition of ZEB1 by small interfering RNA (siRNA) and shRNA

We designed and purchased two different siRNA duplexes of ZEB1, si-ZEB1 (sense, 5′-GCAUCCAAAGAACAAGAAATT-3′) and si-ZEB1-2 (sense, 5′-GCCAACAGUUGGUUUGGUATT, siRNA duplexes (si-Ctrl) (sense, 5′-CAGUCGCGUUUGCGACUGGUU-3′), with the same GC content as ZEB1 siRNA (si-ZEB) from Takara (Tokyo, Japan). The siRNA was used to transfect parental and paclitaxel-resistant EOC cells at a final concentration of 80 nmon/L using GenePorter-2 (Genlantis, San Diego, CA, USA) according to the manufacturer’s protocol.

To generate NOS3TR cells that constitutively expressed the shRNAs, oligonucleotides encoding shRNAs specific for human ZEB1 and luciferase were cloned into the pSIREN-RetroQ vector (Clontech Laboratories, Inc.). The sequences of the shRNAs were: 5′-GCATCCAAAGAACAAGAAA-3′ (shZEB1) and 5′-CTTACGCTGAGTACTTCGA-3′ (control). Recombinant retrovirus was produced, and infected NOS3TR cells were selected with 1 μg/mL puromycin for 3 days.

### PTX chemosensitivity assay

The PTX chemosensitivity assay was performed as described previously [37]. Briefly, cells were seeded in triplicate in 96-well plates at a density of 5,000 cells in a volume of 200 μL of culture media containing 10% FBS. After incubation for 24 hrs at 37°C, the medium was replaced with fresh medium with or without various concentrations of PTX (Bristol Myers Squib, Tokyo, Japan). After an additional 72 hrs, the cell viability was assayed using the Cell Titer 96 Aqueous One Solution Cell Proliferation Assay kit (Promega Corp., Tokyo, Japan).

### *In vitro* migration and invasion assay

Cell invasion was evaluated using 24-well Matrigel invasion chambers (Becton Dickinson Labware, Franklin Lakes, NJ, USA). Cells were suspended in the upper chamber at a final concentration of 50 × 10^5^/mL in 200 μL of RPMI 1640 supplemented with 0.1% bovine serum albumin (BSA). The lower chamber contained 750 μL of RPMI 1640 supplemented with 10% FBS. After 10 hrs of incubation, the remaining tumor cells on the upper surface of the filters were removed by wiping with cotton swabs, and the invading cells on the lower surface were stained with May-Grűnwald Giemsa staining. Cells on the lower surface of the filters were counted under a microscope at a magnification of 200, and we performed four individual experiments using the invasion assay in triplicate.

Cell migration was assayed in 24-well Transwell cell culture chambers (Costar, Corning, NY, USA). Cells were suspended in the upper chamber at a final concentration of 1.0 × 10^5^/mL in 200 μL of RPMI 1640 medium. The subsequent procedures were the same as those used for the ‘Invasion Assay’. In addition, we examined the effect of siRNA transfection on the migration of parental and PTX-resistant EOC cells. Cells transfected with siRNAs were seeded onto the upper chamber and allowed to migrate to the fibronectin-coated lower surface for 24 hrs. The number of cells that had migrated to the lower surface was counted to evaluate the migratory ability. Cells were seeded in 10-cm dishes in RPMI1640 containing 10% FBS. After reaching 50% confluency, the medium was replaced by fresh RPMI 1640 containing 10% FCS, and transfection of siRNA (si-Ctrl and si-ZEB) was performed using GenePorter-2 (San Diego, CA, USA). Sixty hours after transfection, the cells were trypsinized and pelleted. Subsequently, the cells were re-plated in the upper chambers of Transwell plates at a density of 50 × 10^4^ /mL in 200 μL of RPMI 1640. The lower chamber contained 700 μL of RPMI 1640 supplemented with 10% FBS. The subsequent procedure was the same as described above. We performed four individual experiments in which this assay was performed in triplicate.

### Adhesion assay of monolayered HPMCs

HPMCs were trypsinized from 75 cm^2^ flasks and plated on clear-bottom 96-well microtiter Plates (Falcon, Corning, NY, USA) at 5,000 cells/well. After confluency, the monolayered HPMCs were washed twice with 200 μL of serum-free RPMI 1640 medium. Then, cells were plated at a density of 1.0 × 10^4^ cells/200 μL volume of serum-free RPMI 1640 medium in each well. Culture plates were centrifuged at 500 rpm for 30 sec, and incubated at 37°C for 2 hrs in a humidified atmosphere of 5% CO_2_. After incubation, the plates were washed three times with the assay buffer to remove non-adherent cells. Then, adherent cells were evaluated for cell-viability using a modified tetrazolium salt MTT assay with the Cell Titer 96 Aqueous One Solution Cell Proliferation Assay kit (Promega Corp., Tokyo, Japan). The adhesion index was calculated using the following formula:

Adhesion index={Absorbance(492 nm)of transfected cells plated well)−Absorbance(492 nm)of HPMCs alone}.

The assay was replicated six times for each condition.

### Western blot analysis

The Western blotting procedure was described previously [38]. As primary antibodies, we used anti-E-cadherin (Santa cruz, CA, USA), anti-N-cadherin (Santa Cruz, CA, USA), anti-β-catenin (Santa Cruz, CA, USA), anti-Fibronectin (Santa Cruz, CA, USA), anti-vimentin (Santa Cruz, CA, USA), anti-ZEB1 (Cell Signaling, MA, USA), anti-ERK1 (Santa Cruz, CA, USA), anti-p-ERK1 (Santa Cruz, CA, USA), anti-Akt (Cell Signaling, MA, USA), anti-p-Akt (Cell Signaling, MA, USA), anti- PARP (Cell signaling, MA, USA), anti-cleaved PARP (Cell Signaling, MA, USA), anti-Caspase 9 (Cell Signaling, MA, USA), anti-cleaved Caspase 9 (Cell Signaling, MA, USA), anti-TGF-βR-I (Santa Cruz, CA, USA), anti-p-Smad2 (Cell Signaling, MA, USA), anti-Smad 2 (Cell Signaling, MA, USA), anti-Smad4 (Cell Signaling, MA, USA), and anti-β-actin antibodies (Sigma-Aldrich, MO, USA). The primary antibodies were washed in 0.05% Tween-20/PBS and then incubated with horseradish peroxidase-conjugated secondary antibody. Proteins were visualized using enhanced chemiluminescence reagent (Amersham Pharmacia Biotech, NJ, USA). Bands were visualized by ImageQuant LAS 4000 (GE Company, Fairfield, CT, USA).

### Immunofluorescence experiment

Cells were grown in chamber slides (Nalge Nunc International, Rochester, NY, USA). They were fixed for 15 min with 4% paraformaldehyde and washed several times with PBS. Coverslips were incubated in blocking solution containing 2% BSA in PBS for 1 hr, and incubated with the appropriate primary antibodies {anti-mouse E-cadherin and anti-rabbit β-catenin antibodies (Santa Cruz Biotech, CA, USA)} for 1 hr at room temperature. After incubations with appropriate secondary antibodies (E-cadherin, goat anti-mouse IgG-FITC; β-catenin, goat anti-rabbit rhodamine-conjugated IgG: Santa Cruz Biotech, CA, USA), fluorescence was visualized by epifluorescence con-focal microscopy (Pascal, BIORAD, (Hercules, CA, USA).

### Treatment with TGF-β

NOS2, NOS3, NOS2TR, and NOS3TR cells were stimulated with recombinant human transforming growth factor-β1 (TGF-β1) (R&D Systems, Minneapolis, MN, USA) at 10 ng/mL in RPMI-1640 supplemented with 2% FBS for 96 hrs.

### *In vivo* studies

Female nude mice (BALB/c) at 6 weeks of age were obtained from Japan SLC (Nagoya, Japan). The treatment protocol followed the guidelines for animal experimentation adopted by Nagoya University. NOS3TR parental, sh-Ctrl-transfected, and sh-ZEB-transfected cells (7 × 10^7^ cells /0.5 mL of medium/mouse) were injected i.p. to induce the peritoneal metastasis of EOC in the mouse model. The survival time was examined with or without treatment with PTX. In the PTX administration protocol, the intraperitoneal (i.p.) administration of PTX (20 mg/kg body weight), was initiated 48 hrs after tumor inoculation, and repeated once a week a maximum of 3 times. Control mice was administered of PBS only. Survival times were compared among these two groups, respectively.

### Tunnel assay

Apoptotic cells were identified using the *In Situ* Cell Death Detection kit, Fluorescein (Roche Applied Science, Mannheim, Germany), according to the manufacturer's instructions.

### Statistical analysis

All data are expressed as the mean ± SD. Data were calculated from at least three independent experiments. The significance of differences was analyzed by Student’s *t*-test. A value of *P* < 0.05 was considered to be significant.

## SUPPLEMENTARY MATERIALS FIGURES



## Abbreviations

EMT: (epithelial-mesenchymal transition)
EOC: (epithelial ovarian carcinoma)
Paclitaxel: (PTX)
