# Supplementary material for: Inhibition of ZEB1 leads to inversion of metastatic characteristics and restoration of paclitaxel sensitivity of chronic chemoresistant ovarian carcinoma cells
Source: Oncotarget. 2017 Aug 10;8(59):99482–94. doi: 10.18632/oncotarget.20107 (PMC5725108; doi:10.18632/oncotarget.20107)
Supplement: Supplementary file 1 [file oncotarget-08-99482-s001.pdf]

Inhibition of zeb1 leads to inversion of metastatic characteristics and restoration of paclitaxel sensitivity of chronic chemoresistant ovarian carcinoma cells

SUPPLEMENTARY MATERIALS

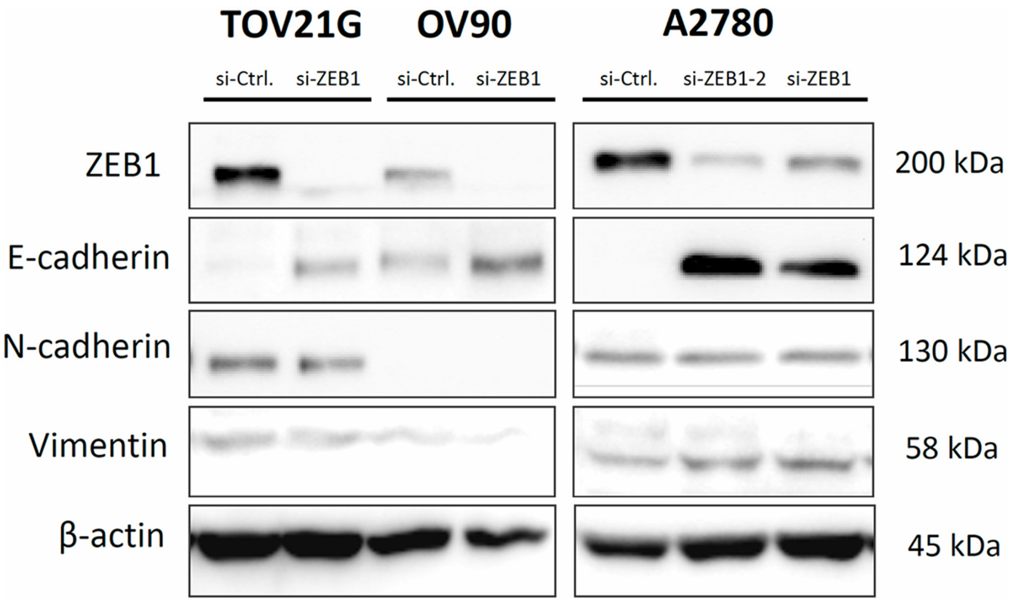

Supplementary Figure 1: Depletion of ZEB1 in TOV-21G, OV90, and A2780 cells and influence on expression of E-cadherin as an epithelial marker, and vimentin and N-cadherin as mesenchymal markers.

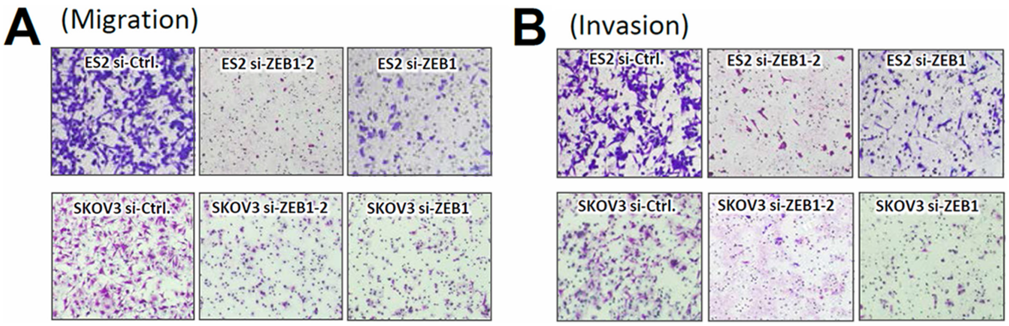

Supplementary Figure 2: The representative images of the decreased cell migration and invasion by depletion of ZEB1 in ES-2 cells.

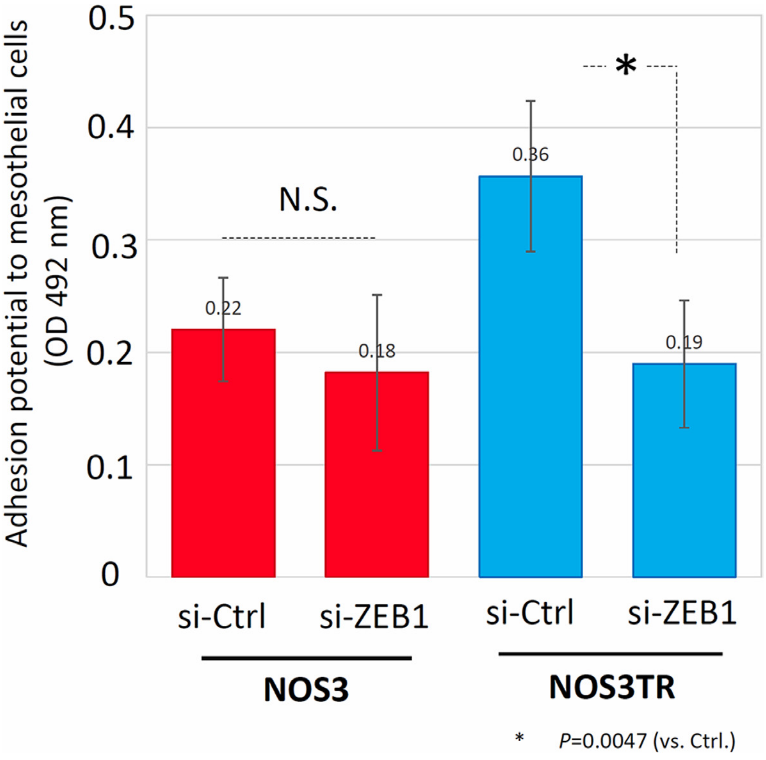

Supplementary Figure 3: The effect of ZEB1 silencing on the adhesion potential of either NOS3 or NOS3TR cells to HPMCs. Asterisks shows statistically significant ($P < 0.05$).
